# The effect of positive parenting on adolescent life satisfaction: the mediating role of parent-adolescent attachment

**DOI:** 10.3389/fpsyg.2023.1183546

**Published:** 2023-07-03

**Authors:** Mengge Li, Ruiming Lan, Peng Ma, Huoliang Gong

**Affiliations:** ^1^School of Psychology, South China Normal University, Guangzhou, Guangdong, China; ^2^School of Psychology, Fujian Normal University, Fuzhou, Fujian, China; ^3^School of Psychology, Henan University, Kaifeng, Henan, China

**Keywords:** adolescent, positive parenting, father’s positive parenting, mother’s positive parenting, life satisfaction, parent-adolescent attachment

## Abstract

This study explores the impact of positive parenting on adolescents’ life satisfaction and the mediating role of parent-adolescent attachment, based on the family systems theory and attachment theory. The sample included 5,047 adolescents (2,353 males, 2,694 females) with a mean age of 16.65 (SD = 1.21) from Henan Province, China. This study used the Positive Parenting Scale, the Inventory of Parent and Peer Attachment and the Satisfaction with Life Scale to survey 5,047 adolescents. The results showed that: (1) father’s positive parenting positively predicted adolescent life satisfaction, while mother’s positive parenting did not significantly predict adolescent life satisfaction; (2) Father-adolescent attachment and mother-adolescent attachment play a mediating role in the relationship between fathers’ positive parenting, mothers’ positive parenting, and adolescent life satisfaction, respectively; (3) Differences in the mechanisms of father’s positive parenting and mother’s positive parenting on adolescent life satisfaction. Among them, mothers’ positive parenting positively predicts mother-adolescent attachment, which in turn affects adolescent life satisfaction. On the other hand, fathers’ positive parenting can influence adolescent life satisfaction through two pathways: by positively predicting father-adolescent attachment and by positively predicting mother-adolescent attachment. The research findings indicate that father’s and mother’s positive parenting have different direct effects on adolescent’s life satisfaction, and both can indirectly influence adolescent life satisfaction through the mediating variables of father-adolescent and mother-adolescent attachment. These findings have important theoretical and practical implications for promoting family education and adolescent psychological well-being.

## 1. Introduction

With the rise of positive psychology, discovering and cultivating positive psychological qualities in individuals has become a hot topic in current academic research. Life satisfaction, as an important indicator for assessing the positive development of individuals’ psychological states (Soares et al., 2019), refers to the comprehensive and overall cognitive evaluation of one’s own quality of life based on self-established standards (Povedano-Diaz et al., 2020). Research has found that life satisfaction among adolescents generally shows a downward trend, and this decline is more rapid than at any other time during adulthood (Orben et al., 2022). The level of life satisfaction among adolescents is closely associated with factors such as academic engagement, self-esteem, and individual psychological well-being (Li, 2018; Freire and Ferreira, 2020; Marcionetti and Rossier, 2021). Therefore, it is necessary to explore the factors and mechanisms that influence adolescent life satisfaction, to provide empirical research support for improving their life satisfaction and promoting their mental health.

### 1.1. Theoretical framework

In the model of adolescent life satisfaction, parenting, as a family parenting environment that is crucial to adolescent psychological development, has an important influence on adolescent life satisfaction and mental health development (Perez-Fuentes et al., 2019). Among them, the study of the impact of parents’ positive parenting behaviors or attitudes on adolescent psychological well-being is currently a focal point of researchers (Liu et al., 2021; Zietz et al., 2022). Based on attachment theory, parenting behaviors affect the quality of the attachment relationship between parents and children. The internal working model formed by good attachment leads to more positive perceptions of self and others, which leads to more satisfying evaluations of life (Bowlby, 1977; Hou et al., 2018). Thus, parent-adolescent attachment relationships may also be an important bridge for positive parenting to influence adolescent life satisfaction. However, there is a relative lack of research based on the attachment theory perspective to explore the mechanisms by which positive parenting influences adolescent life satisfaction.

Furthermore, according to the “spillover” and “crossover” effects of family systems theory (Zou et al., 2019), positive parenting by fathers or mothers may not only “spillover” to affect their own attachment relationships with adolescents, but also “crossover” to affect the attachment relationships between the spouses and the adolescents. Previous research has predominantly focused on overall indicators of positive parenting or the individual effects of fathers and mothers on adolescent individual development (Gherasim et al., 2017; Wang et al., 2022), neglecting to explore the interactive mechanisms between positive fathering and positive mothering in relation to adolescent life satisfaction within the same family system. In contrast, studying a broader range of interactions involving both fathers and mothers with their children can provide a more authentic representation of family dynamics and offer deeper insights into the influence of parents on the psychological well-being of their children (Chan and Leung, 2020).

Therefore, this study aims to bridge the research gap by utilizing family systems theory and attachment theory to explore intact family systems. It will comprehensively examine the influence of positive parenting by both fathers and mothers, as well as father-child and mother–child attachment, on adolescents’ life satisfaction within the same family. These variables will be integrated into a single model to provide a comprehensive understanding of the mechanisms involved. The findings of this study will contribute empirical evidence to enhance relevant theories and provide valuable guidance for counseling practices aimed at improving adolescents’ life satisfaction.

### 1.2. Positive parenting and adolescent life satisfaction

Positive parenting refers to a warm and supportive relationship between parents and children, which serves as a beneficial protective and supportive factor in individual development (Chakroun-Baggioni et al., 2021; Liu et al., 2021). It is noteworthy that there may be a link between fathers’ and mothers’ positive parenting. Zhou et al. (2016) conducted a study on the relationship between parenting styles of fathers and mothers in 896 Chinese adolescent families. They found that positive parenting by fathers and mothers can mutually reinforce each other and jointly promote the positive development of adolescents. Additionally, Tang and Li (2022) conducted a study on 1,379 Chinese adolescent families and found that there is a significant positive correlation between the positive parenting of fathers and mothers. The higher the level of positive parenting by fathers, the higher the level of positive parenting by mothers.

Positive parenting, an important family factor, is predictive of adolescent life satisfaction. Several studies have shown a positive relationship between positive parenting (e.g., warmth, support) and life satisfaction (Wang et al., 2022). At the same time, some scholars have separately explored the effects of fathers’ positive parenting and mothers’ positive parenting on adolescent life satisfaction. The results show that both fathers’ positive parenting and mothers’ positive parenting have their own independent and significant effects on adolescent life satisfaction (Di Maggio and Zappulla, 2014; Gherasim et al., 2017). Clearly, existing research has primarily focused on examining overall indicators of positive parenting or separately investigating the impacts of fathers’ and mothers’ positive parenting on adolescent satisfaction. However, there is a lack of studies that simultaneously examine the effects of fathers’ and mothers’ positive parenting within the same family on adolescent life satisfaction. This gap in research may often overlook the combined effects of positive parenting by both fathers and mothers within the same family. Thus, one of the purposes of this study was to include fathers’ positive parenting, mothers’ positive parenting, and adolescent life satisfaction in the same model to explore the effects of fathers’ positive parenting and mothers’ positive parenting on adolescent life satisfaction within the same family. Family systems theory also states that the attitudes and behaviors of both fathers and mothers, as Co-parenting agents of adolescents, can have a significant impact on the emotional and cognitive development of individual adolescents (Li et al., 2021; Li and Gong, 2022). Based on this, this study proposes hypothesis 1: fathers’ positive parenting and mothers’ positive parenting can positively predict adolescent life satisfaction.

### 1.3. The mediating role of parent–adolescent attachment

Parent–child attachment is a profound, lasting and strong emotional bond between parents and children (Groh et al., 2017). Ainsworth (1979) argued that a child’s attachment characteristics and the quality of attachment depend heavily on the parenting characteristics of the primary caregiver, such as parenting style. A considerable amount of research has explored the significant impact of parental parenting styles on parent-adolescent relationships. Studies have shown that parents’ emotionally supportive and democratic parenting styles contribute to effective communication and the establishment of a positive parent–child attachment relationship (Gregory et al., 2020). On the other hand, adopting authoritarian or rejecting parenting styles can reduce the trust and willingness to communicate between parents and adolescents, leading to conflicts and distancing in the parent–child relationship (Han and Shek, 2012). Furthermore, Liang et al. (2022) have argued that positive parenting by parents fosters a sense of warmth and support, creating a conducive family environment that facilitates the development of a healthy parent–child attachment relationship. A meta-analysis found a positive correlation between positive parenting and children’s secure attachment (Van Ijzendoorn et al., 2023). From the aforementioned research, it becomes apparent that positive parenting by either father or mother can reduce parent–child conflicts, facilitate effective communication, and enhance parent–child attachment relationships. Furthermore, a small number of recent studies have found that the parenting behaviors of fathers and mothers not only influence their own parent–child relationships with adolescents but may also have a “cross-over” effect on the parent–child relationships between spouses and adolescents. For instance, a recent study by Xu et al. (2022), employing a subject-object interdependence model, revealed that parental parenting styles not only predicted the level of intimacy or conflict in their own parent–child relationships but also had the potential to influence the parent–child intimacy or conflict experienced by their spouses. This finding highlights the interconnectedness of different subsystems within families, as posited by family systems theory. Therefore, positive parenting practices by fathers or mothers can positively predict the quality of their own parent-adolescents attachment and may also have a cross-influence on the parent-adolescents attachment quality.

Moreover, there may be a close connection between parent-adolescent attachment and adolescents’ life satisfaction. Guo et al. (2017) pointed out that adolescents with secure attachment tend to have a more positive evaluation and perception of various relationships in their learning and daily lives, leading to a greater experience of happiness and higher satisfaction with life and interpersonal relationships. Furthermore, research has demonstrated that positive parent-adolescent attachment predicts adolescents’ interpersonal relationships, emotional and behavioral adaptation, and levels of psychological well-being (Mónaco et al., 2019; Teng et al., 2020; Hou et al., 2022). Although direct evidence supporting the relationship between parent–child attachment and adolescents’ life satisfaction is currently lacking, we can infer from the aforementioned studies that a higher level of parent–child attachment quality is beneficial for enhancing adolescents’ positive cognition and evaluation of life, improving their psychological well-being, and enhancing their psychological quality. These factors are important variables that promote adolescents’ life satisfaction (Freire and Ferreira, 2020; Gallego et al., 2021; Hu et al., 2023). Based on this, the present study proposes Hypothesis 2: Positive paternal parenting will influence adolescents’ life satisfaction through father-child attachment and also through mother–child attachment. Similarly, positive maternal parenting will influence adolescents’ life satisfaction through mother–child attachment and may also affect adolescents’ life satisfaction through father-child attachment.

**Figure 1:**
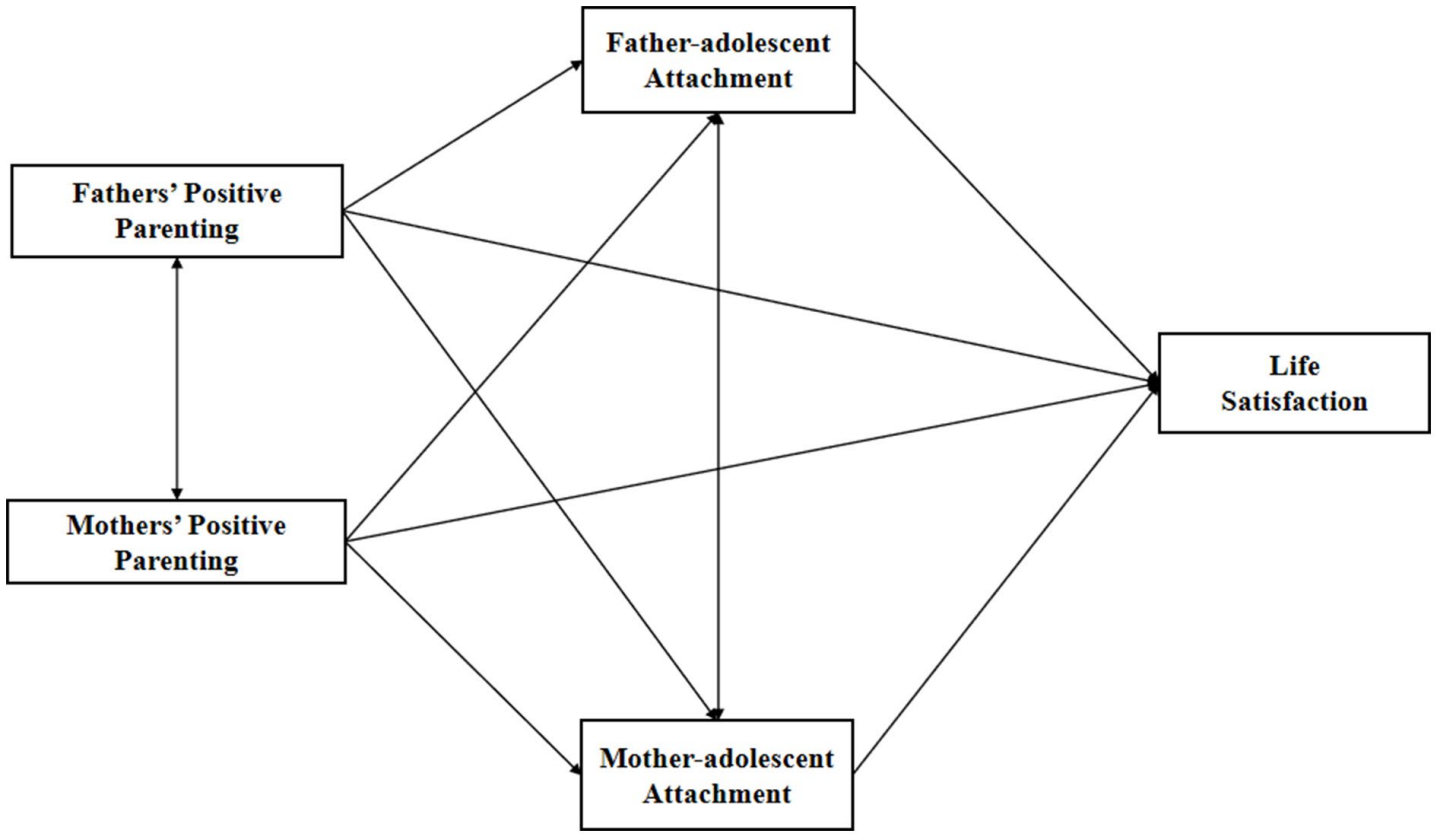
A hypothetical model.

In summary, based on family systems theory and attachment theory, this study proposes a hypothetical model of father’s and mother’s positive parenting affecting adolescent life satisfaction (as shown in the Figure 1) in an attempt to explore the following questions: (1) The impact of father’s positive parenting and mother’s positive parenting on adolescent life satisfaction; (2) The mediating role of parent-adolescent attachment in the relationship between positive parenting and adolescent life satisfaction; (3) Compare the mechanisms of father’s positive parenting and mother’s positive parenting in influencing adolescent life satisfaction.

## 2. Methods

### 2.1. Participants and procedures

Participants were selected from 66 classes in a high school in Henan Province, China, resulting in a total sample of 5,047 teenagers (males = 2,353; females = 2,694). The age of the participants ranged from 14 to 20 years old, with a mean age of 16.65 (*SD* = 1.21). After contacting the school and obtaining the consent of the school and the adolescents, the class teacher will take the students into the computer classroom of the school after negotiating the investigation time with the school. A trained psychology graduate student served as the main test, telling the students how to turn on computer and fill out the questionnaire. The experimenter will also inform the students that they are free to quit the experiment at any time. If they have any questions, they can raise their hands and be answered by the experimenter. Students can leave the classroom after completing the questionnaire and submitting it.

### 2.2. Measurements

#### 2.2.1. The positive parenting subscale

Positive parenting was measured using the 14-item Emotional Warmth subscale from the Chinese version of the Egna Minnen Beträffande Uppfostran, developed by Perris et al. (1980) and validated by Li et al. (2012). There were seven items for the father’s positive parenting style (e.g., “My father always tries to encourage me and make me the best”) and seven items for the mother’s positive parenting style (e.g., “My mother praises me”). Participants scored the items on a 4-point scale from 1 (almost never) to 4 (always), with higher scores indicating more positive parenting behaviors by the parents. The Cronbach α coefficient of the father subscale is 0.92, the average variance extracted (AVE) value is 0.62. The fit index of confirmatory factor analysis is good (χ^2^/*df* = 21.69; CFI = 0.98; TLI = 0.97; SRMR = 0.02; RMSEA = 0.06). The Cronbach α coefficient of the mother subscale is 0.94, the average variance extracted (AVE) value is 0.70. The fit index of confirmatory factor analysis is good (χ^2^/df = 26.15; CFI = 0.98; TLI = 0.97; SRMR = 0.02; RMSEA = 0.07).

#### 2.2.2. The inventory of parent and peer attachment

We adopted items on father–adolescent attachment and mother–adolescent attachment selected from the Chinese version of Inventory of Parent and Peer Attachment, which was developed by Armsden and Greenberg (1987) and revised by Li et al. (2009). It has 13 items across three dimensions: trust, communication, and alienation (e.g., “My father/mother can tell when I am upset about something.”). The participants responded to the items on a 5-point Likert scale ranging from 1 (strongly disagree) to 5 (strongly agree). Higher scores for the adolescent’s attachment to their parents indicate a better relationship with the parents. The father-adolescent attachment’s Cronbach α coefficient of the scale is 0.92, the average variance extracted (AVE) value is 0.47. The fit index of confirmatory factor analysis is good (χ^2^/*df* = 21.25; CFI = 0.95; TLI = 0.93; SRMR = 0.04; RMSEA = 0.06). Mother-adolescent attachment’s Cronbach α coefficient of the scale is 0.93, the average variance extracted (AVE) value is 0.50. The fit index of confirmatory factor analysis is good (χ^2^/*df* = 16.11; CFI = 0.96; TLI = 0.94; SRMR = 0.06; RMSEA = 0.03).

#### 2.2.3. The satisfaction with life scale

The Satisfaction with Life Scale, developed by Diener et al. (1985), consists of 5 items scored on a 7-point Likert scale. Higher scores indicate higher levels of satisfaction. Research results with Chinese participants also indicate that the SWLS demonstrates good reliability and validity in cross-cultural contexts (Leung and Leung, 1992; Xie et al., 2011). The Cronbach α coefficient of this scale is 0.86, the average variance extracted (AVE) value is 0.55. The fit index of confirmatory factor analysis is good (χ^2^/*df* = 25.08; CFI = 0.99; TLI = 0.97; SRMR = 0.02; RMSEA = 0.07).

### 2.3. Data analysis

In this study, all analyses were conducted using SPSS 25.0 and Mplus 8.3. Initially, we performed a Common Methods Variance analysis to address any potential methodological biases. Subsequently, we presented descriptive and correlation analyses. Confirmatory factor analysis (CFA) was also employed to validate the structure of the scales. Notably, Mplus 8.3 was utilized for conducting the structural equation modeling analysis.

## 3. Results

### 3.1. Common methods variance

The Harman’s single factor has been used to analysis the common methods variance. The results show that there are six factors of eigenvalues greater than one, among them the explanatory rate of the first factor is 39.65%, which lower than the criterion of 40%. The results demonstrate that the Common Methods Variance is not obvious. The result is close to the threshold, the reason could be the high correlation between father-adolescent attachment and mother-adolescent attachment, it also high correlation between fathers’ positive parenting and mothers’ positive parenting. The high correlation indicates that the variables could have a same trend, so those could easily lead to CMV. Therefore, it is meaningful that constrain correlation between them in model.

### 3.2. Descriptive analysis and correlation analysis

The results show that (see Table 1) all variables are positively correlated (all *p* < 0.01). In particular, the correlation between all variables is 0.37 to 0.73.

**Table 1:** Descriptive analysis and correlation matrix (*n* = 5,047).

Index	M ± S.D.	1	2	3	4
Age	16.65 ± 1.22	1			
Father-adolescent attachment	49.04 ± 8.76	0.07**	1		
Mother-adolescent attachment	52.10 ± 8.46	0.05**	0.64**	1	
Fathers’ positive parenting	18.68 ± 4.99	0.06**	0.73**	0.51**	1
Mothers’ positive parenting	21.27 ± 4.91	0.05**	0.46**	0.70**	0.65**

^**^p < 0.01, the same below.

### 3.3. Mediation effect

All variables were normalized before formal analysis. Mplus8.3 has been used to analysis chain mediation effect. The fit of the model is perfect which is a saturation model.

From the model effect, the effect of Life satisfaction on Father-adolescent attachment is significant (*coeff* = 0.14, *Z* = 5.61, *p* < 0.001), and the positive effect on fathers’ positive parenting is significant (*coeff* = 0.21, *Z* = 8.90, *p* < 0.001); the life satisfaction also has a positive significant effect on mother-adolescent attachment (*coeff* = 0.20, *Z* = 8.46, *p* < 0.001), but the life satisfaction on mothers’ positive parenting is not significant (*coeff* = 0.03, *Z* = 1.21, *p* > 0.05). Then, the positive effect of father-adolescent attachment on fathers’ positive parenting is significant (*coeff* = 0.74, *Z* = 57.69, *p* < 0.001), but it on mothers’ positive parenting is not significant (coeff = −0.02, *Z* = −1.20, *p* > 0.05). Of course, the positive effect of mother-adolescent attachment on fathers’ positive parenting is significant (*coeff* = 0.09, *Z* = 5.94, *p* < 0.001), and it’s also a significant effect on mothers’ positive parenting (*coeff* = 0.65, *Z* = 47.21, *p* < 0.001). The results are presented in Table 2.

**Table 2:** Chain mediating effect analysis.

Model	DV	IV	Coeff	*Z*
1	Life satisfaction	Father-adolescent attachment	0.14	5.61^***^
		Fathers’ positive parenting	0.21	8.90^***^
		Mother-adolescent attachment	0.20	8.46^***^
		Mothers’ positive parenting	0.03	1.21
2	Father-adolescent attachment	Fathers’ positive parenting	0.74	57.69^***^
		Mothers’ positive parenting	−0.02	−1.20
3	Mother-adolescent attachment	Fathers’ positive parenting	0.09	5.94^***^
		Mothers’ positive parenting	0.65	47.21^***^

DV, the outcome variable; IV, the predictor variable; ^***^p < 0.001, the same below.

**Figure 2:**
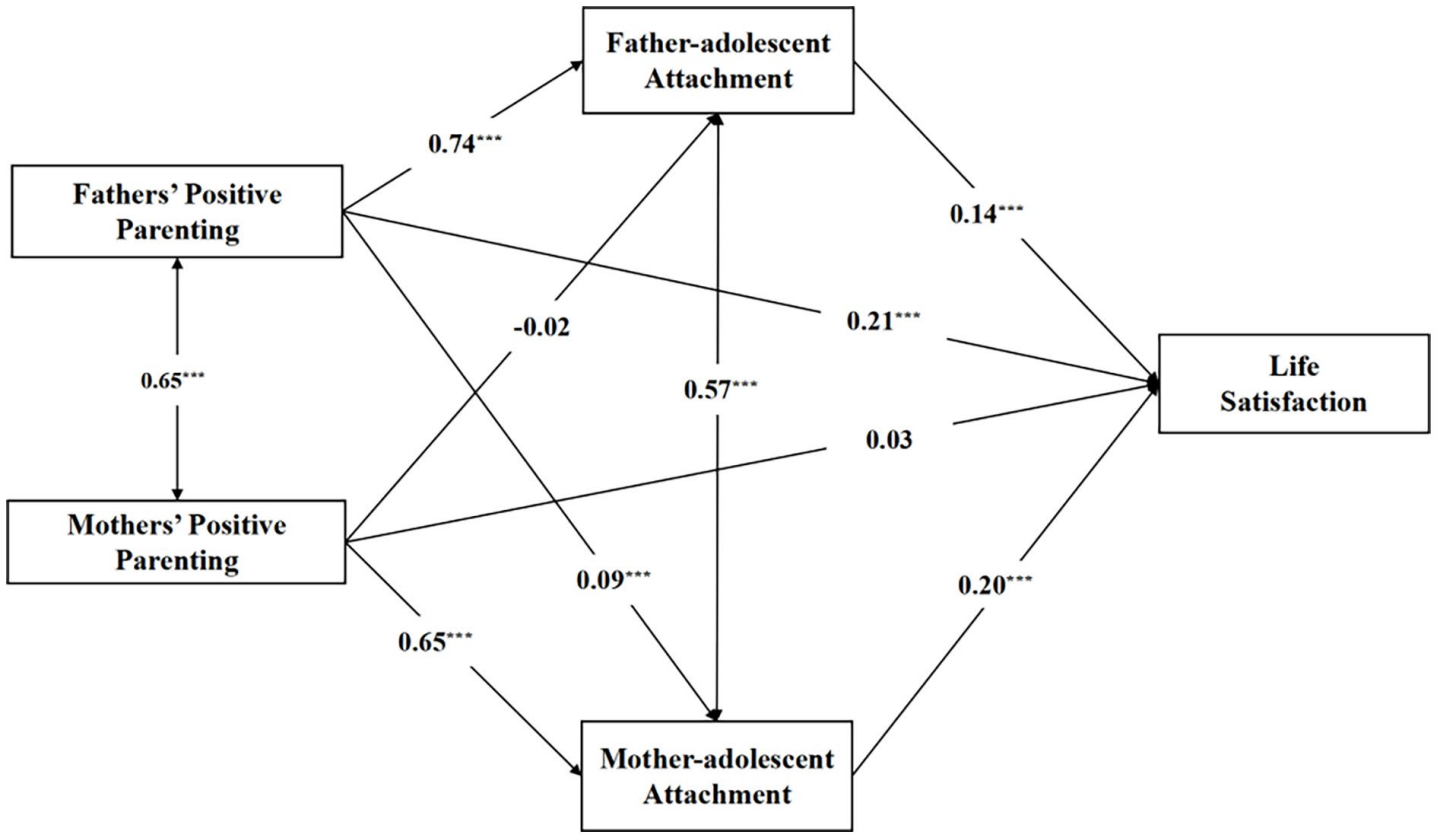
Schematic diagram of a model of life satisfaction.

Further mediation analysis shows that the fathers’ positive parenting and life satisfaction have significant mediation effect between father-adolescent attachment and mother-adolescent attachment; the mother’ positive parenting and life satisfaction also have significant mediation effect between father-adolescent attachment and mother-adolescent attachment (see Figure 2).

In particular, for the effect of fathers’ positive parenting to life satisfaction, the direct effect (0.26) and the total mediation effect (0.14) accounted for 65.00 and 35.00% of the total effect (0.40), respectively (see Table 3). Among the total mediation effect (0.14), the mediation effects of father-adolescent attachment (0.12), mother-adolescent attachment (0.02) which all effects are significant and the 95% confidence interval does not contain 0.

**Table 3:** Total effect, direct effect, and the mediating effect.

	Effect	S.E.	*Z*	Lower 2.5%	Upper 2.5%	Percentage
Direct effect	0.21	0.02	8.90^***^	0.17	0.26	63.64%
Indirect effect	0.12	0.02	6.79^***^	0.09	0.15	36.36%
M1	0.10	0.02	5.59^***^	0.07	0.14	30.30%
M2	0.02	0.01	5.09^***^	0.01	0.03	6.06%
Total effect	0.33	0.02	19.82^***^	0.30	0.37	

M1: the mediating effect of father-adolescent attachment on fathers’ positive parenting and adolescent life satisfaction. M2: the mediating effect of mother-adolescent attachment on fathers’ positive parenting and adolescent life satisfaction. ^***^p < 0.001.

For the effect of mothers’ positive parenting to lift satisfaction, the direct effect (0.03) and the total mediation effect (0.16) accounted for 18.75 and 81.25% of the total effect (0.16; see Table 4). Among the effects, the direct effect and the mediation effect of father-adolescent attachment are not significant.

**Table 4:** Total effect, direct effect, and the mediating effect.

	Effect	S.E.	Z	Lower 2.5%	Upper 2.5%	Percentage
Direct effect	0.03	0.02	1.21	−0.02	0.07	18.75%
Indirect effect	0.13	0.02	7.86^***^	0.10	0.19	81.25%
M1	−0.00	−0.01	−1.16	−0.01	0.00	
M2	0.13	0.02	8.27^***^	0.10	0.16	81.25%
Total effect	0.16	0.02	1.21	0.12	0.19	

M1: the mediating effect of father-adolescent attachment on mothers’ positive parenting and adolescent life satisfaction. M2: the mediating effect of mother-adolescent attachment on mothers’ positive parenting and adolescent life satisfaction.^***^p < 0.01.

Those indicate that the fathers’ positive parenting can not only directly influence life satisfaction, but also can influence life satisfaction through the mediation role of father-adolescent attachment and mother-adolescent attachment. Similarly, mothers’ positive parenting can not only directly influence life satisfaction, but also can influence life satisfaction through the mediation role of mother-adolescent attachment; however, mothers’ positive parenting cannot influence life satisfaction through father-adolescent attachment.

Lower 2.5% and Upper 2.5% refer to the interval estimates of bootstrap2.5 and 97.5%. the same below.

## 4. Discussion

### 4.1. Positive parenting and adolescent life satisfaction

The results of this study found a positive association between fathers’ positive parenting and mothers’ positive parenting, which is consistent with previous findings (Zhou et al., 2016; Tang and Li, 2022). Positive parenting in which one parent adopts a warm, encouraging approach to parenting is more likely to result in positive emotional experiences and positive emotional expressions in children, facilitating positive emotional interactions between family members (Wang et al., 2022). This positive family emotional interaction, in turn, can lead the other parent to choose positive parenting behaviors that are supportive, sensitive, and encouraging (Smith et al., 2007; Wang et al., 2022). Thus, father’s positive parenting and mother’s positive parenting have mutually reinforcing effects.

Additionally, the results of the study showed that father’s positive parenting had a direct positive effect on adolescents’ life satisfaction, supporting the hypothesis 1. This indicates that father’s positive parenting can directly influence adolescent life satisfaction. In the adolescent stage, fathers are more inclined to orient their adolescents to the outside world and teach them more about interpersonal and social adjustment skills, in addition to giving them emotional care (Paquette, 2004). As a result, father’s positive parenting is more likely to directly address adolescents’ growing interpersonal and independence needs, improve social adjustment, and thus enhance life satisfaction.

It is worth noting that the direct effect of mother’s positive parenting on adolescents’ life satisfaction is not significant, which is inconsistent with hypothesis 1. This result also further suggests that fathers’ and mothers’ parenting behaviors play different roles on adolescents’ mental health (Hou et al., 2018; Li et al., 2021). For this result we believe that there are two possible explanations. First, in terms of parenting content, mothers are more likely to provide warm support to their children in terms of living and emotional care, whereas individuals in the adolescent stage are mainly faced with interpersonal and social adjustment problems, and interpersonal relationships are important predictors of adolescent life satisfaction (Man, 1991; Leung and Leung, 1992). Therefore, if mothers do not adjust their parenting content in a timely manner, they will not be able to improve adolescents’ life satisfaction by directly addressing their adolescents’ major concerns. Secondly, in terms of parenting time, in the vast majority of Chinese families, the mother is the primary caregiver for teenagers, which is mainly reflected in the fact that compared to fathers, mothers spend significantly more time in the upbringing of children (Hou et al., 2018). During adolescence, individuals experience heightened self-awareness and a strong desire for independence, leading to a state of imbalance and instability in their psychological development. At this stage, although the mother’s high-frequency and “comprehensive” care and attention can provide adolescents with a sense of warmth and nurturing, the growing need for independence may paradoxically evoke feelings of resentment toward the mother’s excessive attentiveness. Thus, mother’s positive parenting during the adolescent years may not directly influence adolescent life satisfaction.

### 4.2. The mediating role of parent-adolescent attachment in positive parenting on adolescents’ life satisfaction

This study is the first to examine the mediating role of parent–child attachment in the relationship between positive parenting and adolescent life satisfaction. It was found that father’s positive parenting both directly and positively influenced adolescents’ life satisfaction and indirectly through the mediating role of parent-adolescent attachment, which confirmed hypothesis 3. Although mother’s positive parenting does not directly affect adolescents’ life satisfaction, it may indirectly and positively affect adolescents’ life satisfaction through the mediating role of mother-adolescent attachment. The results suggest that father’s and mother’s positive parenting are conducive to improve the quality of parent–child attachment, which in turn promotes adolescents’ life satisfaction. This finding further supports attachment theory (Ainsworth and Bowlby, 1991), as it highlights the significant role of positive parenting in the upbringing of adolescents. Positive parenting fosters effective communication and interaction between parents and adolescents, leading to reduced conflicts and strengthened emotional bonds within the parent–child relationship (Gregory et al., 2020). These positive parent–child attachment relationships contribute to the development of a secure internal working model and a safe haven, providing individuals with feelings of security and warmth. Moreover, they promote the cultivation of self-worth and ultimately enhance the cognitive perception of life satisfaction (Bajaj et al., 2016; Hou et al., 2022). Conversely, parents who use less positive parenting make the adolescent’s experience less supportive, warm interactions, have difficulty developing good internal working models (e.g., the self is important and deserves to be loved) or secure bases, making the individual self-denying or insecure, which greatly reduces the individual adolescent’s life satisfaction.

### 4.3. The differential mechanisms of the positive parenting effects on adolescent life satisfaction between fathers and mothers

This study found that father’s and mother’s positive parenting have different mechanisms of influence on the life satisfaction of adolescents. First of all, mother’s positive parenting affects adolescents’ life satisfaction through “spillover effect.” Specifically, mother’s positive parenting predicts adolescents’ life satisfaction through positive prediction of mother-adolescent attachment. In addition, father’s positive parenting affects adolescent life satisfaction through both a “spillover effect” and a “crossover effect,” as positive fathering predicts adolescent life satisfaction by positively predicting both father-adolescent attachment and mother-adolescent attachment pathways. These results support the “spillover” and “crossover” effects in family systems theory (Zou et al., 2019).

It is noteworthy that there is a unique “crossover effect” of positive fathering on adolescent life satisfaction compared to positive mothering. Specifically, father’s positive parenting influences adolescent life satisfaction by positively predicting mother-adolescent attachment, suggesting that the subsystems in the family system are not independent of each other, but interact through certain patterns. In addition, possible explanations for the existence of unique pathways leading to father’s positive parenting: due to work, family division of labor, and culture, many Chinese mothers spend more time directly involved in child rearing and are the primary caregivers of their children, while at the same time, they also bear a greater stress of child rearing. The father’s involvement in parenting allows for the direct sharing of the mother’s caregiving responsibilities, effectively reducing her parenting burden. This provides the mother with more time and opportunities to engage in harmonious interactions with her child, thereby enhancing the attachment relationship between mother and child (Gao et al., 2020). As a result, positive parenting by both parents uniquely influences adolescent life satisfaction by predicting the mother–child attachment.

### 4.4. Research implications

This study has important theoretical and practical significance for promoting family harmony, improving teenagers’ life satisfaction and improving their mental health. First of all, this study is a revalidation of the family system theory and the attachment theory, and complements the family system theoretical model of the impact of positive parenting on adolescents’ life satisfaction. At the same time, further verification expands the spillover hypothesis and cross hypothesis of family system theory, which is helpful for researchers to understand the family system theory deeply.

Second, this study explores the influence mechanism of adolescent life satisfaction from the perspective of fathers, mothers, and adolescents based on family systems theory and attachment theory from a positive psychology perspective, which helps to inspire future researchers to investigate adolescent mental health issues comprehensively and deeply in the study of adolescent mental health.

Finally, the findings of this study have significant educational guidance implications for parents in enhancing the life satisfaction of adolescents: (1) Encouraging fathers to actively engage in family education, providing support, encouragement, and guidance to promote the life satisfaction of adolescents; (2) Fostering a strong parent–child bond in family education, wherein parents should establish close connections, build trust, and create an emotionally supportive and warm home environment to facilitate the development and consolidation of attachment relationships;(3) Employing diverse parenting approaches in parenting practices, taking into account the unique dynamics between fathers/mothers and their children, as well as the specific needs of adolescents, to formulate appropriate parenting strategies.

### 4.5. Limitations and future directions

Although this study provides theoretical and empirical research support for understanding the mechanisms of how father’s positive parenting and mother’s positive parenting in the family system affects adolescents’ life satisfaction, the following deficiencies remain to be further explored in the future. First, this study is a cross-sectional study, which largely limits the inference of causal relationships among variables. The use of longitudinal studies may be considered in the future to better understand the changing dynamics of the relationship between positive parenting, parent-adolescent attachment and adolescent life satisfaction. Second, the results of this study showed that father’s positive parenting “crossed over” to mother-adolescent attachment, but there is a lack of research evidence as to whether this result was due to the involvement of fathers in reducing maternal parenting stress. Future research could include the variable of maternal parenting stress to provide a more objective basis for the current findings. Finally, this study only explored the mediating role of parent–child attachment, and there may be other mediating variables. More mediating variables could be included in the future to explore the pathways of influence of positive parenting on adolescents’ life satisfaction.

## 5. Conclusion

This study investigates the formation mechanism of adolescents’ life satisfaction from a positive psychology perspective, based on family systems theory and attachment theory. The findings show that father’s positive parenting can significantly and positively influence adolescents’ life satisfaction, while mother’s positive parenting cannot directly influence adolescents’ life satisfaction; father-adolescent attachment and mother-adolescent attachment play a mediating role between positive parenting and adolescent life satisfaction.

## Data availability statement

The raw data supporting the conclusions of this article will be made available by the authors, without undue reservation.

## Ethics statement

The studies involving human participants were reviewed and approved by the Research Ethics Committee of the Institute of Psychology and Behavior, Henan University. Written informed consent to participate in this study was provided by the participants’ legal guardian/next of kin.

## Author contributions

ML conceived the research. RL designed the research. PM performed the research and analyzed the data. ML, RL, and HG contributed to the writing of the manuscript. All authors contributed to the article and approved the submitted version.

## Funding

This research was funded by the Graduate Research and Innovation Fund of the School of Psychology, South China Normal University, for the academic year 2022-2023, grant number PSY-SCNU202211. In addition, this study was supported by the 2021 Henan Provincial Philosophy and Social Science Planning Annual Project——Study on the influence and intervention of academic Autobiographical memory on adolescent learning adjustment (Project No.: 2021BJY004).

## Conflict of interest

The authors declare that the research was conducted in the absence of any commercial or financial relationships that could be construed as a potential conflict of interest.

## Publisher’s note

All claims expressed in this article are solely those of the authors and do not necessarily represent those of their affiliated organizations, or those of the publisher, the editors and the reviewers. Any product that may be evaluated in this article, or claim that may be made by its manufacturer, is not guaranteed or endorsed by the publisher.
